# Case of rapid aortic remodeling after thoracic endovascular aortic repair for retrograde type A aortic dissection

**DOI:** 10.1093/jscr/rjac050

**Published:** 2022-03-01

**Authors:** Yuta Kikuchi, Masahiro Tsutsui, Kohei Ishido, Masahiko Narita, Ryohei Ushioda, Tomonori Shirasaka, Natsuya Ishikawa, Hiroyuki Kamiya

**Affiliations:** Department of Cardiovascular Surgery, Asahikawa Medical University, Asahikawa, Hokkaido, Japan; Department of Cardiovascular Surgery, Asahikawa Medical University, Asahikawa, Hokkaido, Japan; Department of Cardiovascular Surgery, Asahikawa Medical University, Asahikawa, Hokkaido, Japan; Department of Cardiovascular Surgery, Asahikawa Medical University, Asahikawa, Hokkaido, Japan; Department of Cardiovascular Surgery, Asahikawa Medical University, Asahikawa, Hokkaido, Japan; Department of Cardiovascular Surgery, Asahikawa Medical University, Asahikawa, Hokkaido, Japan; Department of Cardiovascular Surgery, Asahikawa Medical University, Asahikawa, Hokkaido, Japan; Department of Cardiovascular Surgery, Asahikawa Medical University, Asahikawa, Hokkaido, Japan

## Abstract

The proper surgical strategy for retrograde type A aortic dissection (RTAD) is still controversial, and some studies have reported the efficacy of frozen elephant trunk and thoracic endovascular aortic repair (TEVAR). A 68-year-old man was diagnosed with acute type A aortic dissection using enhanced computed tomography. The false lumen at the arch and ascending aorta was thrombosed, and the primary entry was placed in the descending aorta. In addition, there were malperfusions of the right renal artery and both iliac arteries. We performed TEVAR using the right femoral artery combined with the petticoat technique. At 11 days postoperatively, we observed rapid aortic remodeling at the arch and ascending aorta. The patient was discharged uneventfully after 14 days. We believe that TEVAR for RTAD is effective in appropriate patients. However, the accumulation of the number of cases and accurate strategies for patient selection are in demand.

## INTRODUCTION

Retrograde type A aortic dissection (RTAD) is a subgroup of type A aortic dissection (TAAD) in which entry is placed at the descending aorta, involving ~7% of all TAADs [1]. The main operative strategy for aortic dissection is the exclusion (or resection) of the primary entry [2]. However, the strategy for the treatment of RTAD remains controversial because of the entry position. Some have reported cases treated with frozen elephant trunk (FET; [3, 4]) or thoracic endovascular aortic repair (TEVAR; [5, 6]), but there are not enough cases to evaluate its efficacy. Here, we report a case of rapid aortic remodeling after TEVAR for RTAD. TEVAR is a promising strategy for the treatment of RTAD, especially for malperfusion cases, due to its minimally invasive nature and rapid increase in true lumen blood flow.

## CASE REPORT

**Figure 1:**
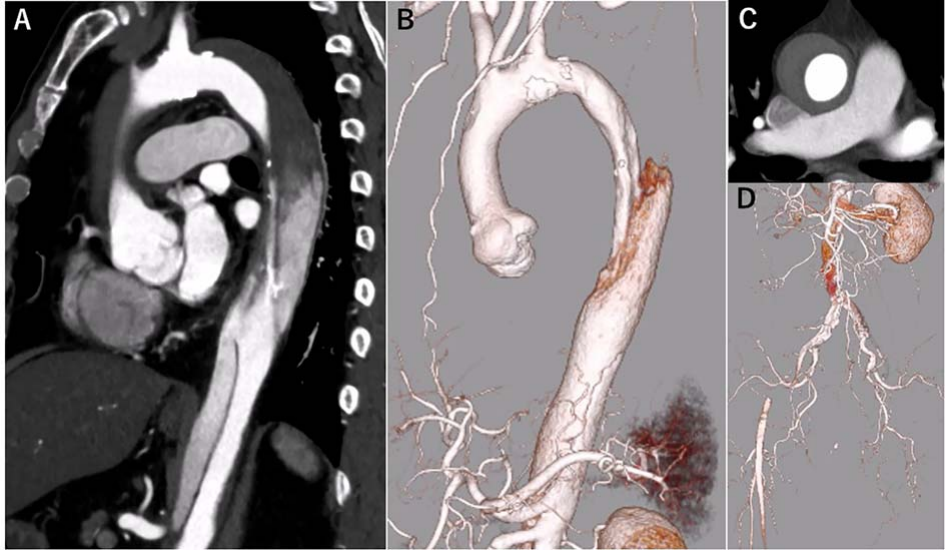
eCT at diagnosis. (**A**): Sagittal view, (**B**) 3D reconstruction of the ascending and descending aorta, (**C**): Axial view of the ascending aorta and (**D**): 3D reconstruction of the abdominal aorta and iliac artery.

A 68-year-old man developed severe acute back pain and was diagnosed with acute type A aortic dissection with enhanced computed tomography (eCT) in the emergency department of our institute (Fig. 1A and B). The false lumen of the arch and ascending aorta was thrombosed (the maximum short diameter of the ascending aorta was 43.3 mm, the maximum diameter of the false lumen at the ascending aorta was 17.7 mm, Fig. 1C), and the primary entry was placed at the descending aorta. In addition, malperfusions were observed in the right renal artery and both iliac arteries (Fig. 1D). Therefore, we decided to perform TEVAR with the petticoat technique because of the rapid increase in true lumen blood flow.

**Figure 2:**
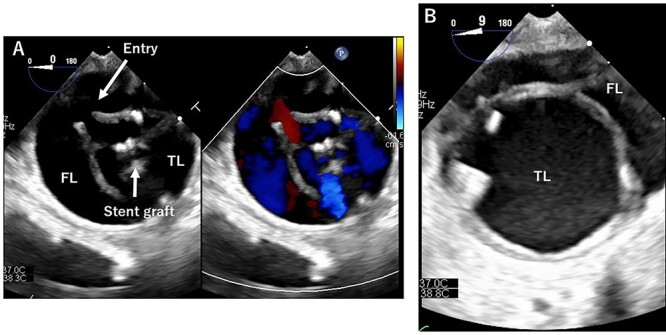
TEE during the operation. (**A**): At entry site. Entry and stent grafts are shown. FL, false lumen; TL, true lumen. (**B**): Expansion of the TL.

**Figure 3:**
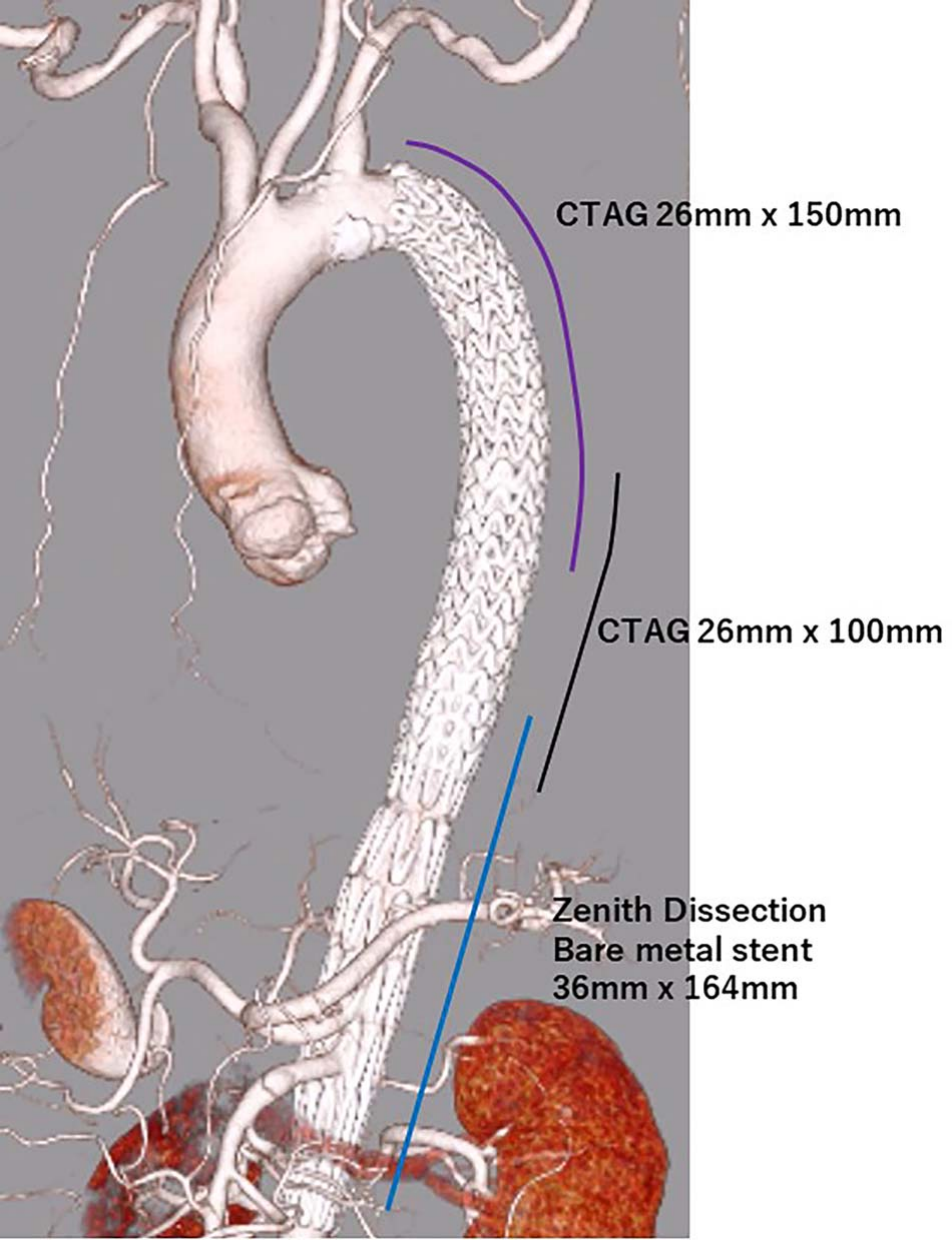
eCT at 1-day postoperatively.

TEVAR was performed through the right femoral artery (rFA) with cut-down. Pulsation was not observed; therefore, we confirmed that the rFA was not dissected using echography from the surface. A 5Fr sheath (Medikit Super Sheath, Medikit, Tokyo, Japan) and 0.035-inch guidewire (Radifocus, Terumo, Tokyo, Japan) were inserted carefully, and we confirmed it in the true lumen using transesophageal echography (TEE) during the operation (Fig. 2A). Subsequently, a 5Fr pigtail catheter (Medikit, Tokyo, Japan) was inserted, and the guidewire was changed to a stiff wire (Lunderquist extra-stiff guidewire, Cook, Bloomington, IN, USA). Then, a 20 Fr Dryseal sheath (WL Gore and Associates, Flagstaff, AZ) was inserted. Through the sheath, we deployed Conformable TAG (26 mm × 100 mm; WL Gore and Associates, Flagstaff, AZ) at the descending aorta. Next, we deployed Conformable TAG (26 mm × 150 mm; WL Gore and Associates, Flagstaff, AZ) just distal to the left subclavian artery to the first stent graft with sufficient overlap. At this point, we observed expansion of the true lumen with TEE (Fig. 2B). Finally, we placed the Zenith Dissection Bare stent (36 mm × 164 mm; Cook, Bloomington, IN) at the distal end of the second stent graft with one stent overlap (Fig. 3). We confirmed the improvement of malperfusion and no access trouble.

**Figure 4:**
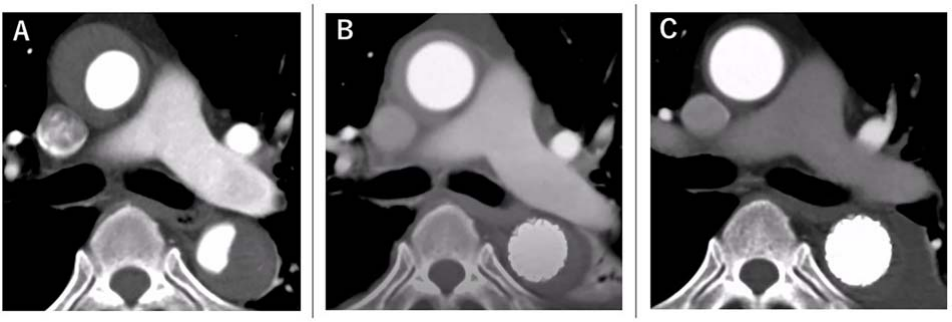
The course of aortic remodeling at the ascending aorta. (**A**): Preoperative, (**B**): 1-day postoperatively, (**C**): 11-day postoperatively (see Video 1).

**Figure 5:**
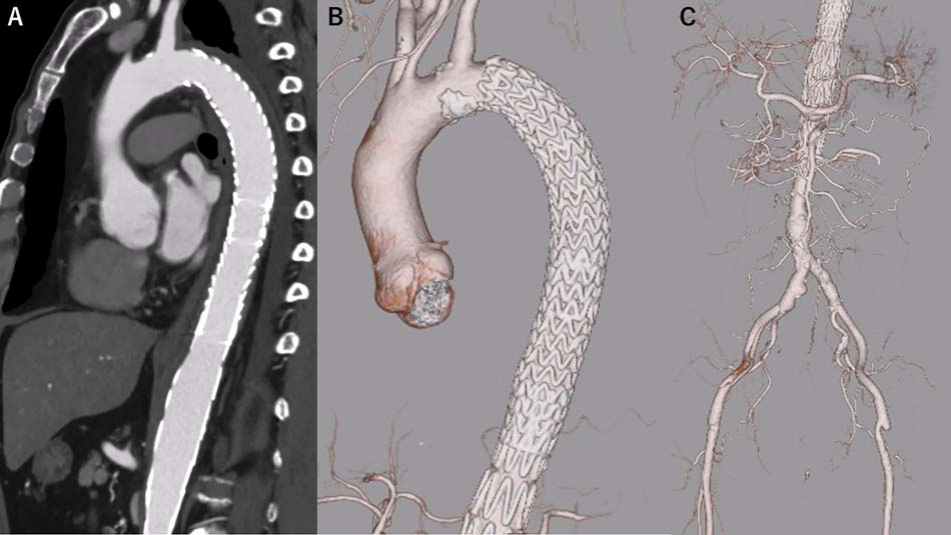
eCT at 11 days postoperatively. (**A**): Sagittal view, (**B**, **C**): 3D reconstruction of the aorta.

The postoperative course was uneventful, and he was discharged 14 days postoperatively. Rapid aortic remodeling was observed with eCT at 1 and 11 days postoperatively (Fig. 4). Right iliac artery dissection did not impede blood flow and no other complications such as stent-induced new entry (SINE) were observed (Fig. 5). We strictly followed up the false lumen and iliac dissection.

## DISCUSSION

In the present report, rapid aortic remodeling occurred successfully without any complications. However, Higashigawa *et al*. reported that the 5-year survival rate was 93%, but the reintervention related to dissection or TEVAR was 42%. Therefore, they said that TEVAR is a good option for RTAD in terms of ‘survive’, however, a strict follow-up is needed [6].

TEVAR is relatively easy to perform, but the strategy for aortic dissection is complicated regarding the selection of the device, its size and access route. In addition, TEVAR landing at the dissected aorta is a risk factor for stent-induced RTAD at the proximal landing, so we also have to consider the landing zone appropriately. In our case, we used CTAG because it has active control that can be aligned with short curvature and prevents bird-beak phenomenon at the proximal landing zone [7]. We believe that the device deployed half of it at first, and then fully, so the impact on the intima of the dissected aorta was mitigated.

The size of the stent graft tends to be small if the true lumen is small. In this case, we used 26 mm, which is the smallest we could use in an emergency, but it is a bit large. An oversized stent graft is a risk factor for stent-induced RTAD; therefore, smaller devices are mandatory in dissection cases.

If both femoral access routes are occluded due to dissection, we consider other access routes or we must confirm that the guidewire is in the true lumen. In our institute, TEE was conducted to confirm the wire position and status of the true or false lumen. Intravascular ultrasonography has also been reported in the literature [8]. Regardless of the method used, we must confirm the guidewire in the true lumen, especially the access route that was dissected.

FET has also been reported to be an effective method for the treatment of RTAD [3, 4]. However, there are some complications such as paraplegia, misdeployment into the false lumen and distal SINE [9–11]. In addition, this procedure was conducted with open repair; therefore, invasiveness was higher, and rapidity was lower than in TEVAR. Therefore, if applicable, TEVAR should be considered for appropriate patients.

In summary, we report a case in which rapid aortic remodeling was successfully observed after TEVAR for RTAD. TEVAR is one of the most important options for the treatment of RTAD; however, the long-term results are unknown. The patient selection criteria for TEVAR for RTAD have not yet been established. Because it is uncommon, sufficient cases do not accumulate for analysis. Therefore, further studies are required.

## Supplementary Material

video_1_rjac050
